# When Menstruation Meets the Pleura: A Rare Case of Catamenial Hemothorax and Thoracic Endometriosis and a Literature Review

**DOI:** 10.7759/cureus.72496

**Published:** 2024-10-27

**Authors:** Bouchra Daher, Fadila Kouhen, Oussama Afandi, Youssef Mahdi, Najiba Yassine

**Affiliations:** 1 Pulmonology, Cheikh Khalifa International University Hospital, Mohammed VI University of Sciences and Health (UM6SS), Casablanca, MAR; 2 Radiotherapy, Cheikh Khalifa International University Hospital, Mohammed VI University of Sciences and Health (UM6SS), Casablanca, MAR; 3 Thoracic Surgery, Cheikh Khalifa International University Hospital, Mohammed VI University of Sciences and Health (UM6SS), Casablanca, MAR; 4 Pathology, National Institute of Oncology of Rabat, Rabat, MAR

**Keywords:** catamenialhemothorax, hormone therapy, pleural effusion, thoracic endometriosis syndrome, videoassisted thoracoscopy

## Abstract

Catamenial hemothorax is a rare manifestation of thoracic endometriosis, characterized by blood in the pleural cavity associated with menstrual cycles. We present the case of a 42-year-old woman with recurrent right-sided chest pain and dyspnea coinciding with menstruation. Imaging revealed a large pleural effusion and hemothorax. Diagnostic video-assisted thoracoscopic surgery (VATS) confirmed thoracic endometriosis. The patient received a combination of hormonal therapy and VATS. Follow-ups over 36 months showed significant symptom improvement and resolution of pleural effusion. This case emphasizes the need to consider thoracic endometriosis in women with menstrual-related hemothorax.

## Introduction

Hemothorax is a rare and often unexpected manifestation that can reveal underlying thoracic endometriosis [1]. Thoracic endometriosis syndrome (TES) is defined by the presence of functional endometrial tissue within the thoracic cavity, including the lungs, pleura, diaphragm, or bronchial system [2]. This condition primarily affects women of reproductive age and presents with a range of clinical symptoms, often linked to the menstrual cycle [3].

Diagnosing TES is particularly challenging due to its nonspecific symptoms and the rarity of the condition. The pathophysiology of TES is not completely understood, but several theories exist. These include retrograde menstruation with subsequent transdiaphragmatic migration of endometrial cells, coelomic metaplasia, and lymphatic or hematogenous spread of endometrial tissue from the pelvis to the thorax [4].

Clinical presentation can vary widely depending on the location of the endometrial tissue. Common manifestations include catamenial pneumothorax, hemothorax, pleural effusion, and pulmonary nodules, with symptoms typically worsening during menstruation. Radiological imaging, such as chest X-rays and CT scans, along with video-assisted thoracoscopic surgery (VATS), play crucial roles in diagnosing TES. Histopathological examination of biopsy samples remains the gold standard for definitive diagnosis [5].

The management of TES requires a multidisciplinary approach that combines hormonal therapy to suppress ovarian function and surgical intervention to remove endometrial implants and repair diaphragmatic defects [6]. Hormonal treatments often involve the use of gonadotropin-releasing hormone (GnRH) agonists, danazol, or progestins [7].

We present a case of catamenial hemothorax revealing thoracic endometriosis, treated with hormonal therapy and VATS, along with a comprehensive review of the existing literature to elucidate optimal treatment approaches.

## Case presentation

**Figure 1:**
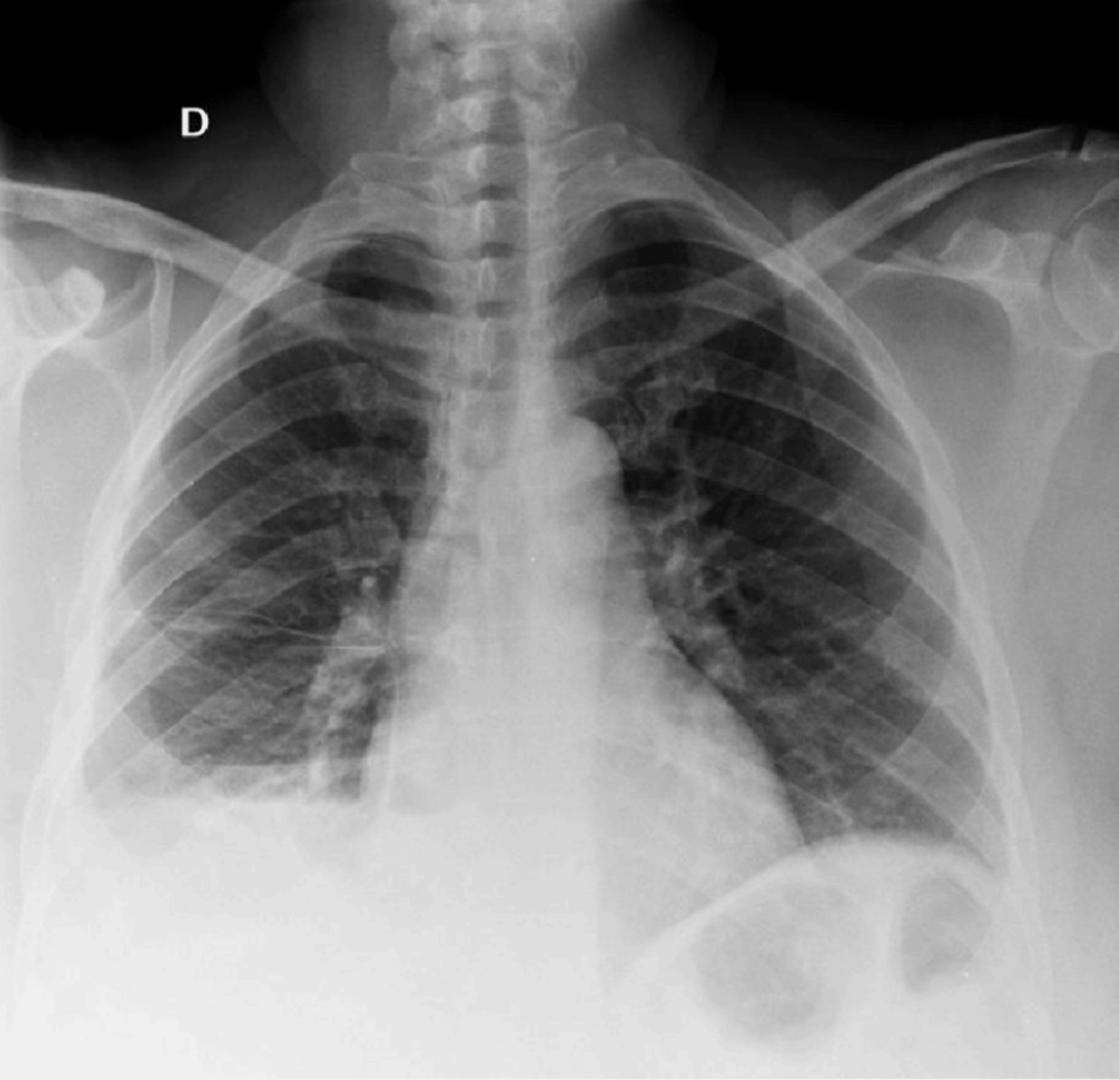
Chest X-ray demonstrating moderate pleural effusion in the right lung, indicated by blunting of the right costophrenic angle.

This case concerns a 42-year-old female of Caucasian origin, married and a mother of one, with no significant past medical history. Since 2013, she has experienced recurrent, paroxysmal abdominopelvic pain correlating with her menstrual cycle, managed symptomatically as dysmenorrhea. In March 2023, during a diagnostic workup for secondary infertility, 18 years post-delivery, laparoscopy revealed pelvic endometriosis. Concurrently, the patient developed a four-month history of severe right-sided pleuritic chest pain, disturbing her sleep and resistant to standard analgesics. This symptom was associated with stage 2 modified Medical Research Council (mMRC) dyspnea, cough and hemoptysis, accompanied by a marked deterioration in her overall health, including an 8 kg weight loss over the same period. A chest X-ray demonstrated pleural effusion (Figure 1).

Thoracentesis revealed a serohematic, exudative pleural effusion with a predominance of lymphocytes on cytological analysis.

Cytological analysis of pleural fluid was performed to assess the nature of the pleural effusion and to exclude the possibility of tuberculosis and malignancy. The results were inconclusive, showing no signs of tuberculosis, such as caseating granulomas or acid-fast bacilli, and no cancerous cells were identified. 

Further biological workup revealed moderate inflammatory syndrome and hypochromic microcytic anemia, characterized by a hemoglobin level of 10 g/dL (normal range: 12.1 - 15.1 g/dL), a red blood cell count of 3.5 million cells/μL (normal range: 4.2 - 5.4 million cells/μL), a mean corpuscular volume of 70 fL (normal range: 80 - 100 fL), a ferritin level of 20 ng/mL (normal range: 11 - 307 ng/mL), and a C-reactive protein level of 10 mg/L (normal: < 10 mg/L). Negative serologies for hepatitis B virus (HBV), hepatitis C virus (HCV), and HIV were also noted, along with a normal CA-125 level of 30 U/mL (normal: < 35 U/mL).

Given the clinical suspicion of tuberculous pleuritis based on the patient's symptoms and the hemothorax, empirical anti-tuberculosis therapy was initiated despite the lack of bacteriological or histological confirmation.

**Figure 2:**
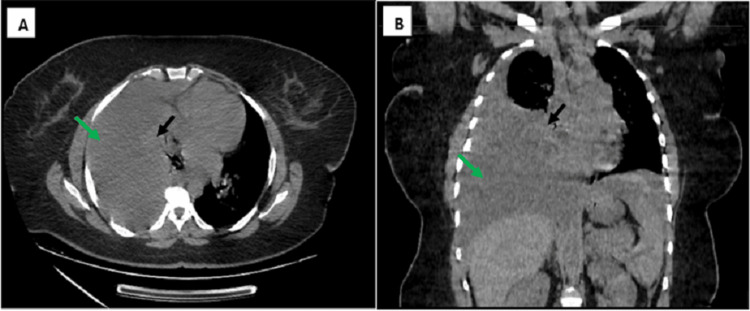
Computed Tomography (CT) in the mediastinal window illustrating a large hemothorax (green arrow) with associated lung collapse (black arrow): A. Axial slice and B. Coronal reconstruction.

Despite initial improvement, the patient subsequently experienced four episodes of recurrent pleurisy, coinciding with menstruation. A thoracic CT scan revealed a large pleural effusion, associated lung collapse, an apical pneumonia focus on the right side, and perihepatic and perisplenic effusions, indicating a significant deterioration in her condition (Figure 2).

**Figure 3:**
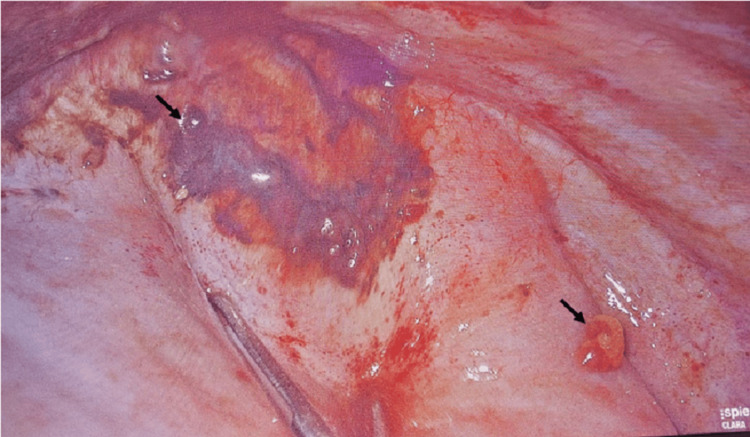
Endometriotic Pleural Lesions Revealed by Video-Assisted Thoracoscopic Surgery (as demonstrated by black arrow).

The decision was made to employ VATS for the diagnostic evaluation and potential treatment of suspected thoracic endometriosis. During the procedure, violaceous nodules were identified on the diaphragm, parietal pleura, and lung parenchyma with a low-volume hemothorax (Figure 3).

Multiple biopsies were obtained, and visible lesions were excised or cauterized to alleviate symptoms and mitigate the risk of recurrence.

**Figure 4:**
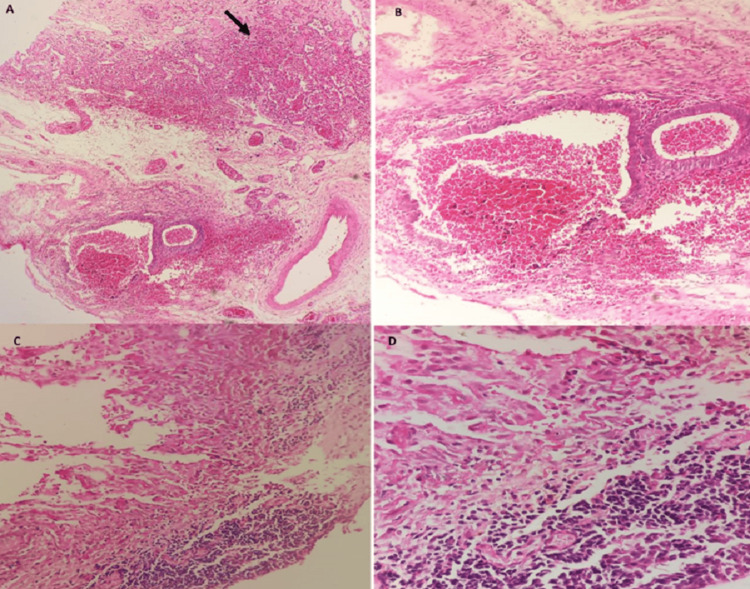
Representative micrographs of the lesion. Lesion is composed of endometrial-type glands, endometrial-type stroma and hemorrhage (A and B) (arrow: pulmonary tissue). In some areas, endometrial-type stroma is the only identifiable component (C and D). (Hematoxylin-eosin; A: ×100, B: x200, C: x200, D: x400).

Analysis of the lung biopsies revealed the presence of endometrial tissue, characterized by both endometrial glands and stroma (Figure 4).

Immunohistochemical studies supported this diagnosis, showing positive staining for estrogen receptors (ER), progesterone receptors (PR), and CD10, which are indicative of endometrial tissue. In contrast, the biopsies from the pleura and diaphragm displayed nonspecific subacute inflammatory changes without the distinctive endometrial features. Although the biopsies of the pleural and diaphragmatic nodules showed nonspecific subacute inflammatory changes, they supported the diagnosis of localized inflammation.

Post-surgery, the patient was treated with progestins, aimed at suppressing estrogen production and reducing endometrial tissue growth. 

After the surgical intervention and initiation of hormonal therapy, the patient was scheduled for regular follow-up appointments every three months to closely monitor her progress, focusing on the resolution of her chest pain and dyspnea while identifying any new or recurring issues.

**Figure 5:**
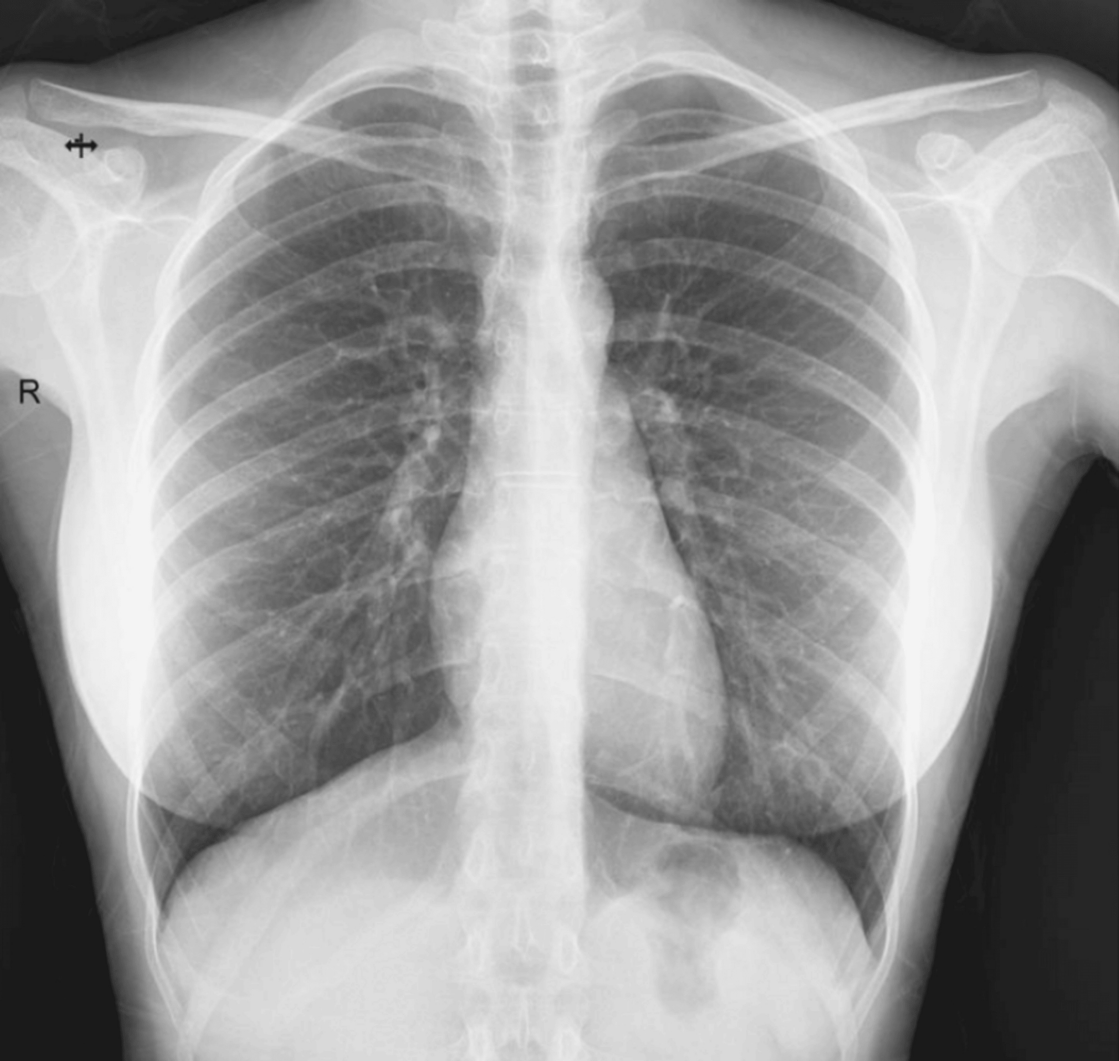
Chest X-rays Following Six Months of Hormonal Treatment Demonstrating No Pleural Effusion, Indicating a Positive Therapeutic Response.

These 36-month follow-ups confirmed a complete resolution of the previously observed pleural effusion as well as lung collapse (Figure 5).

The progestin-based hormonal therapy was found to be effective, resulting in significant symptom relief and no major side effects. The patient also received guidance on lifestyle modifications and symptom management strategies to support her long-term health. 

## Discussion

Thoracic endometriosis is a relatively uncommon condition, affecting approximately 1% of women of reproductive age and is associated with TES. Clinical histories often reveal prior pelvic surgeries, uterine curettage, or a history of infertility [7].

The clinical manifestations of thoracic endometriosis depend on the specific thoracic structures involved. Catamenial pneumothorax is the most prevalent manifestation of TES, occurring in approximately 70% of cases. It predominantly affects the right side, with bilateral involvement being rare and left-sided cases being exceptionally uncommon. Other manifestations include pleural effusion (15%), hemoptysis (7%), pulmonary nodules (6%), diaphragmatic hernia, and chronic thoracic pain [8-10]. These symptoms are typically observed during menstruation, specifically starting the day before and extending up to 72 hours after the onset of menstruation [11].

Hemothorax is a relatively rare manifestation of thoracic endometriosis, with infrequent cases of associated pneumothorax, almost exclusively on the right side. Only one documented instance involves left-sided hemothorax associated with right-sided catamenial pneumothorax. Symptoms of hemothorax are nonspecific and include cough, chest pain, and dyspnea. The volume of effusion can vary, with some cases presenting with hypovolemic shock. In my patient, given the moderate volume of hemothorax, there were no clinical signs indicative of shock.

Conventional chest X-rays are nonspecific, typically demonstrating pleural effusion without further detail [12,13]. Thoracic CT scans may reveal pleural nodular lesions or, rarely, true pleural masses or loculated effusions. MRI is more effective than CT for identifying endometriosis, displaying hyperintense signals on T1- and T2-weighted images [14].

In our case, the patient experienced recurrent right-sided chest pain that correlated with her menstrual cycle. Chest X-ray revealed a significant hemothorax and thoracic CT demonstrated a large hemothorax with associated lung collapse, an apical pneumonia focus on the right, and perihepatic and perisplenic effusions.

From a diagnostic perspective, serum CA-125 levels have been suggested as a useful biomarker, due to their sensitivity and specificity [15]. In our case, the CA-125 level was 30 U/mL, which is within the normal range and may not strongly indicate the presence of thoracic endometriosis. However, cytological examination of pleural fluid rarely yields endometrial cell aggregates. Bronchoscopy is limited in diagnosing endometriosis due to the peripheral location of most lesions [16]. The definitive diagnosis relies on surgical exploration, particularly VATS, which should comprehensively assess the parietal pleura, visceral pleura, lung parenchyma, and diaphragm.

Histopathological confirmation involves the identification of endometrial tissue, including both glands and stroma. Immunohistochemical analysis is crucial in detecting minimal lesions and utilizes markers such as estrogen and progesterone receptors and CD10 to establish the diagnosis [17].

Currently, a multidisciplinary approach combining surgical intervention and hormonal therapy offers the most effective management for thoracic endometriosis, optimizing outcomes by reducing recurrence rates and alleviating chronic symptoms [18]. VATS remains the preferred technique for initial intervention, enabling both the identification and excision of endometriotic lesions, which concurrently provides histopathological confirmation, and is the technique used in our patient [19]. In cases requiring more extensive resections, video-assisted minithoracotomy is advisable to enhance surgical access and precision. Given the high propensity for disease recurrence, pleurodesis using talc is recommended to induce a permanent pleural symphysis and minimize the risk of reaccumulation of pleural effusion. For our patient, we preferred to let it be in case of recurrence of the effusion to assess the patient’s natural response and avoid additional interventions, while closely monitoring their condition. Traditional thoracotomy is reserved for scenarios where VATS proves inadequate or fails [2].

The management of thoracic endometriosis follows similar principles to pelvic endometriosis, focusing on suppressing ovarian estrogen production to control symptoms through medications like GnRH agonists, which are typically used for a maximum of six to 12 months, danazol as a third-line option, and progestins [20,21]. Regular follow-up evaluations every three months are essential to monitor the patient’s response and adjust treatment as needed. Long-term management can be challenging due to limitations on the duration of certain therapies, so continuous oral progestins or hormonal intrauterine devices (IUDs) may be used for symptom control. In severe cases, surgical options, including the removal of endometriosis lesions from the thoracic cavity or a hysterectomy with or without bilateral salpingo-oophorectomy, may be considered, especially for patients nearing menopause, while carefully weighing the risks of surgical menopause [22].

Further research is needed to explore long-term treatment options for endometriosis and identify alternatives that offer sustained symptom management with minimal side effects.

## Conclusions

Thoracic endometriosis is an exceptionally rare condition with limited reported cases. It should be considered in women of reproductive age who present with symptoms exacerbated by menstruation. Diagnosing this condition can be challenging and typically requires histopathological confirmation. Our case underscores the importance of considering this diagnosis in similar presentations. Early diagnosis and a multidisciplinary approach are crucial for optimal treatment outcomes and to mitigate the recurrence of symptoms.

## References

[1] Patrucco Reyes S, Amoah K, Rahi MS, Gunasekaran K (2021). A case of hemothorax as manifestation of thoracic endometrial syndrome. J Investig Med High Impact Case Rep.

[2] Nezhat C, Lindheim SR, Backhus L, Vu M, Vang N, Nezhat A, Nezhat C (2019). Thoracic endometriosis syndrome: a review of diagnosis and management. JSLS.

[3] Azizad-Pinto P, Clarke D (2014). Thoracic endometriosis syndrome: case report and review of the literature. Perm J.

[4] Signorile PG, Viceconte R, Baldi A (2022). New insights in pathogenesis of endometriosis. Front Med (Lausanne).

[5] Joseph J, Sahn SA (1996). Thoracic endometriosis syndrome: new observations from an analysis of 110 cases. Am J Med.

[6] Ciriaco P, Muriana P, Lembo R, Carretta A, Negri G (2022). Treatment of thoracic endometriosis syndrome: a meta-analysis and review. Ann Thorac Surg.

[7] Nezhat C, Main J, Paka C (2014). Multidisciplinary treatment for thoracic and abdominopelvic endometriosis. JSLS.

[8] Adeoye PO, Adeniran AS, Adesina KT (2018). Thoracic endometriosis syndrome at University of Ilorin Teaching Hospital. Afr J Thorac Crit Care Med.

[9] Keijzer S, Oosterhuis W, Hazelbag HM, Meuleman T (2021). Pathological diagnosis of thoracic endometriosis. BMJ Case Rep.

[10] Bagan P, Le Pimpec Barthes F, Assouad J (2003). Catamenial pneumothorax: retrospective study of surgical treatment. Ann Thorac Surg.

[11] Bobbio A, Canny E, Mansuet Lupo A (2017). Thoracic endometriosis syndrome other than pneumothorax: clinical and pathological findings. Ann Thorac Surg.

[12] Jablonski C, Alifano M, Regnard JF, Gompel A (2009). Pneumoperitoneum associated with catamenial pneumothorax in women with thoracic endometriosis. Fertil Steril.

[13] Bobbio A, Carbognani P, Ampollini L, Rusca M (2007). Diaphragmatic laceration, partial liver herniation and catamenial pneumothorax. Asian Cardiovasc Thorac Ann.

[14] Rousset P, Rousset-Jablonski C, Alifano M, Mansuet-Lupo A, Buy JN, Revel MP (2014). Thoracic endometriosis syndrome: CT and MRI features. Clin Radiol.

[15] Herranz-Blanco B, Daoud E, Viganò P, García-Velasco JA, Colli E (2023). Development and validation of an endometriosis diagnostic method based on serum biomarkers and clinical variables. Biomolecules.

[16] Lam S, Shah PL (2021). Bronchoscopic diagnosis of peripheral lung lesions. Respiration.

[17] Camboni A, Marbaix E (2021). Ectopic endometrium: the pathologist’s perspective. Int J Mol Sci.

[18] Lee JH (2024). Commentary: thoracic endometriosis: the necessity of a multidisciplinary approach for optimal treatment. J Chest Surg.

[19] Wetzel A, Philip CA, Golfier F (2021). Surgical management of diaphragmatic and thoracic endometriosis': a French multicentric descriptive study. J Gynecol Obstet Hum Reprod.

[20] Vannuccini S, Clemenza S, Rossi M, Petraglia F (2022). Hormonal treatments for endometriosis: the endocrine background. Rev Endocr Metab Disord.

[21] Surrey ES (2023). GnRH agonists in the treatment of symptomatic endometriosis: a review. F S Rep.

[22] Singh SS, Suen MW (2017). Surgery for endometriosis: beyond medical therapies. Fertil Steril.

